# Cerebral blood flow network differences correlated with cognitive impairment in mild traumatic brain injury

**DOI:** 10.3389/fnins.2022.969971

**Published:** 2022-07-22

**Authors:** Min Duan, Yin Liu, Fengfang Li, Liyan Lu, Yu-Chen Chen

**Affiliations:** ^1^Department of Radiology, Jiangsu Province Hospital of Chinese Medicine, Affiliated Hospital of Nanjing University of Chinese Medicine, Nanjing, China; ^2^Department of Radiology, Nanjing First Hospital, Nanjing Medical University, Nanjing, China

**Keywords:** mild traumatic brain injury, cognitive impairment, arterial spin labeling, cerebral blood flow, magnetic resonance imaging

## Abstract

**Purpose:**

To examine whether the cerebral blood flow (CBF) and CBF connectivity differences are sex-specific and whether these differences are correlated with cognitive impairment in mTBI.

**Methods:**

Resting-state perfusion magnetic resonance imaging was performed in 40 patients with acute mTBI and 40 healthy controls by using pseudocontinuous arterial spin labeling within 14 days following injury. The differences in normalized CBF were first compared and CBF connectivity of the brain regions with significant CBF differences were compared next. The association between the normalized CBF and CBF connectivity differences and cognitive function were further investigated.

**Results:**

Men patients had lower normalized CBF in the frontal gyrus, temporal gyrus and hippocampus and decreased negative CBF connectivity between brain regions including the hippocampus, temporal gyrus, postcentral gyrus and lenticular nucleus, putamen, compared with men controls. Women patients had lower normalized CBF in the frontal gyrus, however had higher normalized CBF in the temporal gyrus and hippocampus, compared with women controls. Additionally, women patients showed increased positive CBF connectivity between the seed region of interest (ROI) of the right inferior temporal gyrus and temporal gyrus and frontal gyrus, and had increased positive CBF connectivity between the seed ROI of the right hippocampus and the temporal gyrus. Furthermore, men patients had higher CBF in the right middle temporal gyrus and left precentral gyrus than women patients.

**Conclusion:**

This study provides evidence of sex differences in both decreased and increased CBF and CBF connectivity and association with cognitive outcome in the acute stage after mTBI.

## Introduction

Mild traumatic brain injury (mTBI) is a significant public health concern, resulting in neurological deficits (Malik et al., 2022). Early prediction of mTBI and recognition of cognitive deficit may improve patient quality of life (Krueger et al., 2021). Hence, it is important to identify mTBI patients at risk or with cognitive deteriorations (Mortaheb et al., 2021).

Sex factor plays a pivotal role in the epidemiology of mTBI, since sex hormone levels, brain cortical thickening, complexity, societal expectations and help-seeking preferences could differ between sexes (Kisser et al., 2016). Clinical measurements of the sex effect on mTBI recovery have been explored, but results were inconclusive. Fakhran et al. (2014) found that men sex and uncinate fasciculus (UF) fractional anisotropy (FA) values are independent risk factor for persistent post-concussion symptoms after 3 months and are stronger predictors of time to symptom resolution. Frommer et al. (2011) found that men patients report more cognitive deficits than women patients. Bai et al. (2019) found only men mTBI patients showed impaired language fluency and cognitive information processing speed, but self-reported symptoms were not much different between sexes. However, female rodents were reported to have better cognitive sequelae in one animal study (Sahbaie et al., 2018) due to neuroprotective effects of female sex steroids.

Interestingly, cerebral blood flow (CBF) which represents one of the most enduring markers of mTBI could be influenced by sex. Substantial mTBI researches have been conducted to study the CBF by using single photo emission computed tomography (SPECT), positron emission tomography (PET), or perfusion weighted imaging (PWI). However, these imaging tools were limited by requirement of exogenous contrast or tracers (Ge et al., 2009; Li et al., 2020).

Arterial-spin labeling (ASL), as a promising MRI approach, could provide reproducible and reliable quantitative measurements of cerebral perfusion non-invasively (Bai et al., 2019; Alisch et al., 2021). This technique has been implemented to detect CBF in mTBI individuals (Bai et al., 2019). Recent mTBI-related functional studies have reported alterations of CBF (Ge et al., 2009; Grossman et al., 2013). Conversely, Bai et al. (2019) showed no significant sex differences of CBF changes in patients with mTBI within 14 days after injury. Neurocognitive deficits have been attributed partly to cerebral hypoperfusion. Alisch et al. (2021) found that CBF differs between men and women. Several human studies to date have demonstrated sex differences in CBF in multifocal brain regions and cognitive outcome in mTBI patients (Stephens et al., 2018; Bai et al., 2019). Accordingly, CBF connectivity is also an important tool which has been applied in autism spectrum disorders (ASD) (Cui et al., 2017), schizophrenia (Jann et al., 2016), and mTBI (Li et al., 2020).

At present, no studies have directly explored sex differences in CBF connectivity in acute mTBI. Accordingly, this study aimed to investigate the sex differences in the alterations of CBF and CBF connectivity in mTBI patients at the acute stage by using pseudo-continuous arterial spin labeling (pcASL) technique. Moreover, the association between CBF and CBF connectivity differences and cognitive dysfunction was also explored. We hypothesized that we would observe different sex impacts on CBF and CBF connectivity in the mTBI group at the acute stage. We also anticipated that the difference of CBF and CBF connectivity may influence cognitive function within the mTBI group. This study could provide a better understanding of the sex difference in early brain perfusion alterations in acute mTBI, and further provide the insight into the underlying pathophysiological mechanism of sex-specific cognitive impairment.

## Materials and methods

### Participants

The protocol of this study was approved by the Ethics Committee of Nanjing Medical University, and all participants provided written informed consent before MRI examination. Between November 2017 and May 2020, patients with a diagnosis of mTBI were prospectively enrolled in this study. MTBI was defined based on the American Congress of Rehabitation Medicine. The matched healthy control participants were recruited through local advertisements.

Inclusion criteria for mTBI patients were as follows: (a) patients aged 18 or older; (b) loss of consciousness < 30 min, Glasgow Coma Score GCS of 13–15 and post-traumatic amnesia < 24 h and time after trauma should be within 14 days, which was defined as acute stage. Exclusion criteria for all subjects were: (a) previous head injury (any history of concussion, mild TBI and moderate to severe TBI); (b) history of cerebrovascular disorders, epilepsy, brain tumors, diabetes and neurological surgery; (c) history of illicit drug use or substance abuse; (d) dental appliances that might distort the functional MR images; (e) left-handed.

The clinical neurocognitive state of all participants was quantified with the Montreal cognitive assessment (MoCA) (Nasreddine et al., 2005) within 24 h after MRI scan by two experienced psychologists, which evaluated seven aspects of cognitive functions, including visuospatial/execution, naming, memory (short-term immediate and deferred recall), language, abstraction, orientation and attention.

### Data acquisition

A 3.0 Tesla MRI scanner (Ingenia, Philips Medical Systems, Netherlands) with an 8-channel head coil was performed in this study. ASL data, high-resolution T1-weighted anatomic imaging data, Fluid-attenuated inversion recovery (FLAIR) and susceptibility weighted imaging (SWI) data were acquired for each subject. The ASL scan lasted for 4 min 08 s. SWI used 3D gradient echo (GRE) sequence. Functional images were obtained axially using a gradient echo-planar imaging sequence as follows: (a) The resting-state perfusion imaging was performed using a pseudocontinuous arterial spin labeling (pCASL) sequence [repetition time (TR) = 4000 ms; echo time (TE) = 11 ms; label duration = 1650 ms; post-label delay = 2000 ms; flip angle (FA) = 90°; field of view (FOV) = 240 mm × 240 mm; slice thickness = 4 mm with 10% gap; matrix = 64 × 64; 20 axial slices; total scan duration = 4 min 08 s]. Finally, each subject contained 60 volumes used as 30 label-control image pairs; (b) Sagittal 3D T1-weighted images were acquired using a three-dimensional turbo fast echo (3D-TFE) T1WI sequence (TR = 8.1 ms; TE = 3.7 ms; FA = 8°; FOV = 256 mm × 256 mm; matrix = 256 × 256; slice thickness = 1 mm, gap = 0 mm; and 172 sagittal slices; total scan duration = 5 min 28 s); (c) SWI: TR = 22 mm; TE = 34 ms; FA = 20; matrix = 276 × 319; slice thickness = 1 mm; FOV = 220 mm × 220 mm.

### Data preprocessing

The pCASL data was processed using Statistical Parameter Mapping (SPM, version 8^1^), ASL data processing toolbox (ASLtbx^2^), and resting-state functional MRI (fMRI) data analysis toolkit (REST^3^). Firstly, ASL images were corrected for head motion. Participants whose translations and rotations were more than 2 mm and 2°, respectively were removed from this study. Secondly, CBF maps were calculated using ASLtbx, each participant’s CBF map was coregistered to their structural image, and individual structural images were normalized in Montreal Neurological Institute (MNI) space; spatial transforms were concatenated to bring the CBF image to MNI template, with resampling to a 2 mm^3^ × 2 mm^3^ × 2 mm^3^ voxel size. Thirdly, the normalized CBF maps were then spatially smoothed with a Gaussian of 8 mm × 8 mm × 8 mm full-width at half maximum (FWHM). The structural MR images were segmented into white matter (WM), gray matter (GM), and cerebrospinal fluid (CSF) using the standard uniform segmentation model. The gray matter volume (GMV) of each voxel was calculated using SPM8 and were taken as covariate for statistical analysis in order to correct the influence of atrophy or partial volume effects (Ashburner, 2007).

### Cerebral blood flow analysis

First, the normalized CBF were compared between groups. Then, the clusters with significant group differences in the CBF were selected as ROIs (Zhu et al., 2015; Shang et al., 2020; Zhang et al., 2020). The CBF values for each ROI within each participant were extracted from a separate CBF map and ROI-based correlation analysis was used to calculate the correlation coefficient (CBF connectivity) between each ROI and all other voxels of the entire brain across individuals for each group. Multiple comparisons correction was performed using the AlphaSim program determined by Monte Carlo (single voxel *p-*value = 0.001, a minimum cluster size of 13, within a GM mask corresponding to the AAL atlas). Then, the CBF connectivity maps for each group were merged into a spatial mask, where each voxel’s normalized CBF is correlated with a normalized CBF of ROI in either group. A specific T comparison was performed within the spatial mask in order to map the voxels which exhibited significantly different normalized CBF correlation with each ROI among groups.

### Statistical analysis

The Kolmogorov–Smirnov test was performed to check the normality of clinical and demographic data distribution. One-way analysis of variance (ANCOVA) and the chi-squared (χ^2^) test was used to investigate the differences among groups. The continuous variables which were not normally distributed were analyzed by Kruskal–Wallis test among groups or by Mann–Whitney U test. SPSS 23.0 software (version 23.0, SPSS Inc., Chicago, IL, United States) was used in above statistical analysis. *P* < 0.05 was considered to be statistically significant.

For the normalized CBF and CBF connectivity analysis, group comparison was performed using ANCOVA, and *post hoc* analysis was performed within any two groups. For *post hoc* analysis, this study used *t*-tests to perform all pairwise comparisons between group means. Then, surface-based cluster-wise correlation for multiple comparisons was performed at the significance threshold of *p* < 0.001 and the cluster-size threshold of 13 mm^2^, which was determined by Monte Carlo (single voxel *p-*value = 0.001, a minimum cluster size of 13, within a GM mask corresponding to the AAL atlas). SPM 8 and REST were used in above statistical analysis. The correlations between normalized CBF and CBF connectivity within significant regions and MoCA score and MoCA sub-score were investigated separately using Spearman correlation analysis in SPSS 23.0 software, and *p* < 0.05 was considered statistically significant. Bonferroni correction for multiple comparisons was performed. The covariates for the above analysis included age, education, and individual mean GMV.

## Results

### Demographic data

Demographics of the subjects were summarized in Tables 1–3. At the emergency department, all patients with mTBI had an initial GCS of 15. All patients had acute CT scans and showed CT negative and no extracranial injury. Causes of head injury included: motor vehicle accidents (MVA) (24), assaults (10), and falls (6). The major causes of injury were a MVA [men: 12/18 (67%), women: 12/22 (55%)], followed by assaults [men: 6/18 of 18 (33%), women: 4/22 (18%)], and falls [men: 3/18 (17%), women: 3/22 (14%)], and for all *p*-value > 0.4 by Chi-square. Neurocognitive data was shown in Tables 1–3. mTBI patients showed significant lower MoCA scores and orientation score (all *p* < 0.05), compared with healthy controls. Compared with women controls, women patients exhibited worse language performance (*p* = 0.005). Differences in age, sex, and education level between mTBI patients and healthy controls were not significant (all *p* > 0.05). mTBI patients showed significant lower MoCA score (*p* < 0.001), compared with healthy controls. No significant differences of age, education level and MoCA scores were found between men and women patients with mTBI. For all the patients, no significant contusion or cerebral hemorrhage were found on routine 3T MRI were found. The symptoms were mild, therefore, all patients take no medications and treatments.

**TABLE 1:** Summary of demographic characteristic between patients with mTBI and healthy controls.

Characteristics	mTBI (*n* = 40)	Controls (*n* = 40)	*P*-value
Age (year)	40.70 ± 10.64 (25–61)	40.50 ± 10.17 (24–65)	0.932
Sex (men/women)	18/22	16/24	0.651
Education (year)	12.98 ± 2.86	13.08 ± 2.91	0.877
GCS Score	15	–	–
Time since injury (day)	2.95 ± 1.61 (0–7)	–	–
MoCA score	24.23 ± 2.37	26.08 ± 1.72	< 0.001
Visuospatial/executive	3.63 ± 1.28	4.15 ± 1.08	0.050
Naming	2.85 ± 0.36	2.85 ± 0.36	1.000
Attention	2.50 ± 1.36	2.93 ± 1.35	0.164
Language	2.33 ± 0.69	2.60 ± 0.55	0.052
Abstraction	1.60 ± 0.59	1.70 ± 0.46	0.402
Memory	5.78 ± 0.42	5.83 ± 0.39	0.582
Orientation	5.53 ± 0.75	5.85 ± 0.43	0.020

Data are presented with the mean ± standard deviation and distribution; mTBI, mild traumatic brain injury; GCS, Glasgow Coma Scale; MoCA, Montreal cognitive assessment.

**TABLE 2:** Detailed demographic characteristics in mTBI patients and healthy controls.

Characteristics	mTBI		*P*-value	Controls		*P*-value
	Women	Men		Women	Men	
Age (year)	42.64 ± 11.88	38.33 ± 8.63	0.207	38.21 ± 9.90	43.94 ± 9.89	0.081
Education (year)	12.45 ± 2.65	13.61 ± 3.05	0.207	12.67 ± 3.13	12.98 ± 2.52	0.938
GCS Score	15	15	–	–	–	–
Time since injury	2.98 ± 1.52	2.96 ± 1.75	0.175	–	–	–
MoCA	23.86 ± 2.61	24.67 ± 2.03	0.292	25.96 ± 1.92	26.25 ± 1.39	0.605
Visuospatial/executive	3.36 ± 1.36	3.94 ± 1.11	0.154	4.00 ± 1.32	4.38 ± 0.50	0.216
Naming	2.86 ± 0.35	2.83 ± 0.38	0.796	2.83 ± 0.38	2.88 ± 0.34	0.726
Attention	2.55 ± 1.30	2.44 ± 1.46	0.819	2.67 ± 1.52	3.31 ± 0.95	0.107
Language	2.27 ± 0.63	2.39 ± 0.78	0.605	2.75 ± 0.44	2.38 ± 0.62	0.052
Abstraction	1.55 ± 0.60	1.67 ± 0.59	0.525	1.75 ± 0.44	1.63 ± 0.50	0.411
Memory	5.73 ± 0.46	5.83 ± 0.38	0.437	5.92 ± 0.28	5.69 ± 0.48	0.099
Orientation	5.50 ± 0.86	5.56 ± 0.62	0.819	5.88 ± 0.34	5.81 ± 0.54	0.656

Data are the mean ± standard deviation; mTBI, mild traumatic brain injury; GCS, Glasgow Coma Scale; MoCA, Montreal Cognitive Assessment.

**TABLE 3:** Demographic characteristics of men and women.

Characteristics	Men		*P*-value	Women		*P*-value
	mTBI	Control		mTBI	Control	
Age (year)	38.33 ± 8.63	43.94 ± 9.89	0.087	42.64 ± 11.88	38.21 ± 9.90	0.175
Sex (number)	18	16	–	22	24	–
Education (year)	13.61 ± 3.05	12.98 ± 2.52	0.938	12.45 ± 2.65	12.67 ± 3.13	0.806
GCS Score	15	–	–	15	–	–
MoCA score	24.67 ± 2.03	26.25 ± 1.39	0.013	23.86 ± 2.61	25.96 ± 1.92	0.003
Visuospatial/executive	3.94 ± 1.11	4.38 ± 0.50	0.150	3.36 ± 1.36	4.00 ± 1.32	0.115
Naming	2.83 ± 0.38	2.88 ± 0.34	0.741	2.86 ± 0.35	2.83 ± 0.38	0.781
Attention	2.44 ± 1.46	3.31 ± 0.95	0.051	2.55 ± 1.30	2.67 ± 1.52	0.774
Language	2.39 ± 0.78	2.38 ± 0.62	0.955	2.27 ± 0.63	2.75 ± 0.44	0.005
Abstraction	1.67 ± 0.59	1.63 ± 0.50	0.828	1.55 ± 0.60	1.75 ± 0.44	0.191
Memory	5.83 ± 0.38	5.69 ± 0.48	0.332	5.73 ± 0.46	5.92 ± 0.28	0.103
Orientation	5.56 ± 0.62	5.81 ± 0.54	0.209	5.50 ± 0.86	5.88 ± 0.34	0.066

Data are the mean ± standard deviation; mTBI, mild traumatic brain injury; GCS, Glasgow Coma Scale; MoCA, Montreal Cognitive Assessment.

### Group differences in normalized cerebral blood flow

**FIGURE 1:**
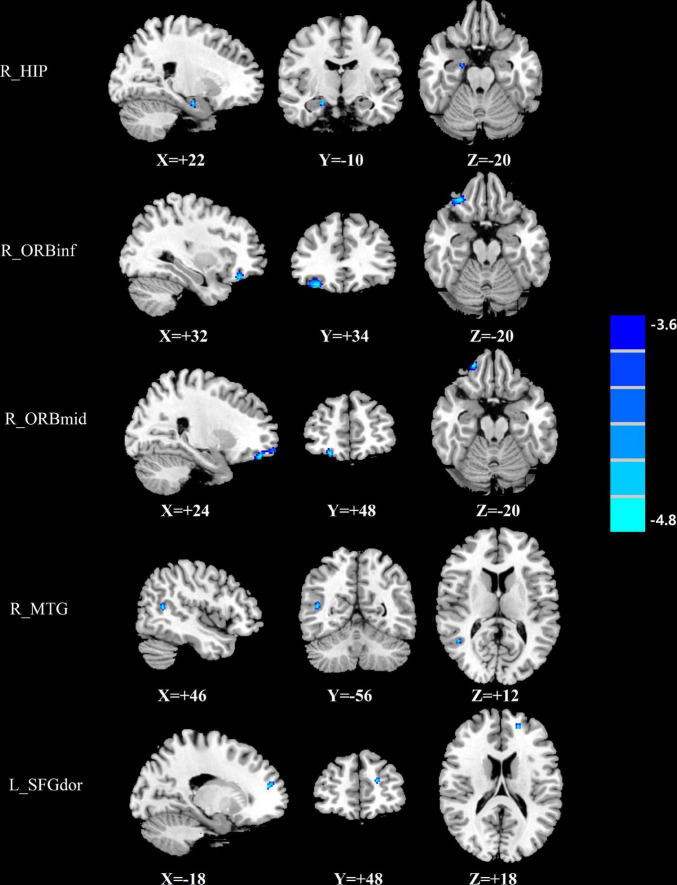
Brain regions showing significant differences in normalized CBF between men patients and men controls. The cold color indicates that the normalized CBF was significantly decreased in the men patients. CBF, cerebral blood flow; R, right; L, left; HIP, Hippocampus; ORBinf, inferior frontal gyrus, orbital part; ORBmid, middle frontal gyrus, orbital part; MTG, middle temporal gyrus; SFGdor, superior frontal gyrus, dorsolateral.

**FIGURE 2:**
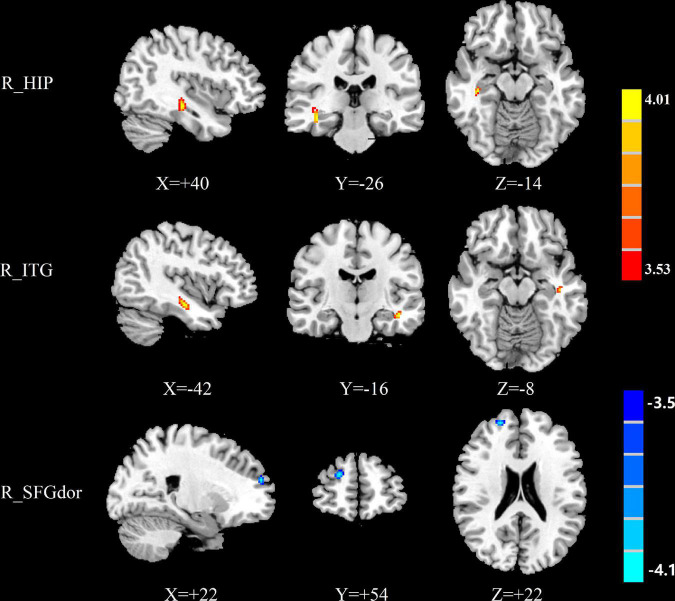
Brain regions showing significant differences in normalized CBF between women patients and women controls. The warm color represents the significantly increased CBF in the women patients. The cold color indicates that the CBF was significantly decreased in the women patients. CBF, cerebral blood flow; R, right; HIP, hippocampus; ITG, inferior temporal gyrus; SFGdor, superior frontal gyrus, dorsolateral.

**FIGURE 3:**
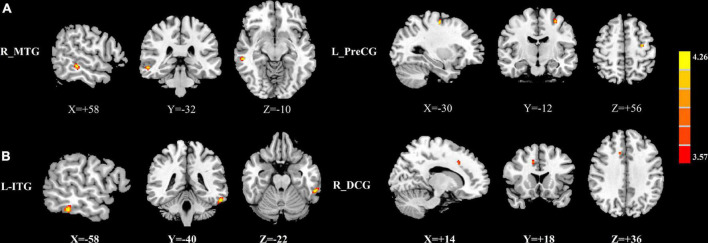
**(A)** Brain regions showing significant differences in normalized CBF between men patients and women patients. **(B)** Brain regions showing significant differences in normalized CBF between men controls and women controls. The warm color represents the significantly increased CBF in the men patients and men controls. CBF, cerebral blood flow, R, right, L, left, MTG, middle temporal gyrus, PreCG, precentral gyrus, ITG, inferior temporal gyrus, DCG, median cingulate and paracingulate gyri.

The group differences in normalized CBF were shown in Figures 1–3 and Table 4. Four groups showed significant differences in normalized CBF in the left cuneus (CUN), right precentral gyrus (PreCG), right postcentral gyrus (PoCG), and left superior temporal gyrus (STG). Men patients showed decreased normalized CBF in the right hippocampus (HIP), right MTG, right inferior frontal gyrus, orbital part (ORBinf), right middle frontal gyrus, orbital part (ORBmid), and left superior frontal gyrus, dorsolateral (SFGdor), compared with men controls (Figure 1). Women patients showed increased CBF in the right ITG and right HIP and decreased CBF in the right SFGdor, compared with women controls (Figure 2). Additionally, among those with mTBI, increased normalized CBF in the right middle temporal gyrus (MTG) and left PreCG was observed in men patients compared with women patients (Figure 3A). Among healthy controls, men controls had higher normalized CBF in the left ITG as well and the right median cingulate and paracingulate gyrus (DCG), compared with women controls (Figure 3B).

**TABLE 4:** Significant brain regions identified for ASL analyses of CBF.

Cohort	Hemisphere	Brain regions	Peak MNI coordinates x,y,z (mm)	*t*-value	Voxels
Four groups	L	CUN	–6,–84,20	21.3304	452
	R	PreCG	20,–18,76	16.9335	197
	R	PoCG	12,–48,78	13.5723	33
	L	STG	–38,–28,–6	13.8450	77
M patients > F patients	R	MTG	58,–32,–10	4.1588	34
	L	PreCG	–30,–12,56	4.1749	21
M controls > F controls	L	ITG	–58,–40,–22	4.4024	65
	R	DCG	14,18,36	3.9421	15
M patients < M controls	R	HIP	22,–10,–20	–4.4302	22
	R	ORBinf	32,34,–20	–5.7592	143
	R	ORBmid	24,48,–20	–5.0749	137
	R	MTG	46,–56,12	–4.2076	27
	L	SFGdor	–18,48,18	–4.0261	31
F patients > F controls	R	HIP	40,–26,–14	4.1751	50
	R	ITG	–42,-16,–8	4.0093	55
F patients < F controls	R	SFGdor	22,54,22	–4.2291	39

M, Men; F, Women; R, Right; L, Left; CUN, Cuneus; PreCG; precentral gyrus; PoCG, postcentral gyrus; STG, Superior temporal gyrus; ITG, Inferior temporal gyrus; MTG, middle temporal gyrus; HIP, Hippocampus; ORBinf, Inferior frontal gyrus, orbital part; ORBmid, Middle frontal gyrus, orbital part; SFGdor, superior frontal gyrus, dorsolateral; DCG, Median cingulate and paracingulate gyri; ASL, Arterial-spin labeling; CBF, cerebral blood flow.

### Group differences in cerebral blood flow connectivity

**FIGURE 4:**
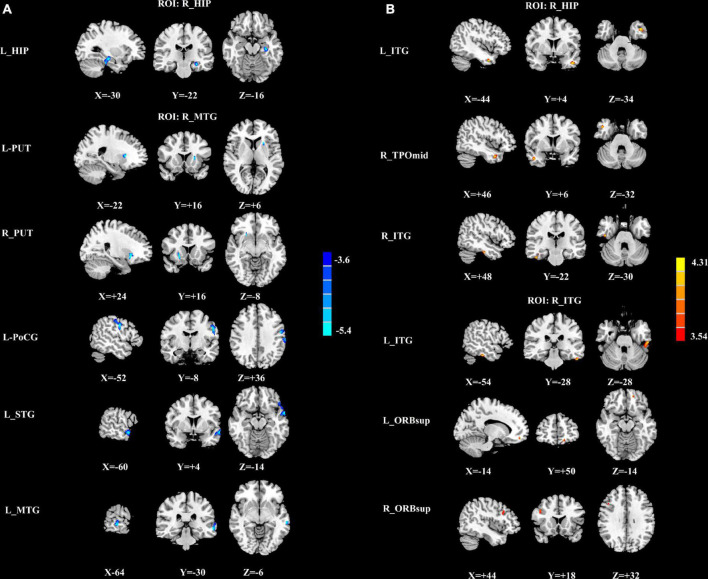
**(A)** The significantly different brain regions of CBF connectivity between men patients and men controls based on the ROI of R_HIP and R_MTG. The cold color indicates that the CBF connectivity was significantly decreased in the men patients. CBF, cerebral blood flow; R, right; L, left; HIP, hippocampus; PUT, putamen; MTG, middle temporal gyrus; STG, superior temporal gyrus; PoCG, postcentral gyrus; **(B)** The significantly different brain regions of CBF connectivity between women patients and women controls based on the ROI of R_HIP and R_ITG. The warm color represents the significantly increased CBF in the women patients. CBF, cerebral blood flow; R, right; L, left; ITG, inferior temporal gyrus; TPOmid, temporal pole: middle temporal gyrus; ORBsup, superior frontal gyrus, orbital part.

Based on normalized CBF results, a total of twelve ROIs, which had significant group differences in normalized CBF, were defined as the seed regions. Group differences in CBF connectivity were shown in Figure 4 and Table 5. Among patients with mTBI, the seed ROI of the right MTG and the left PreCG in CBF did not exhibit any significant differences in the CBF connectivity. Compared with men controls, men patients exhibited decreased negative CBF connectivity between the seed ROI of the right HIP and the left HIP and decreased negative CBF connectivity between the seed ROI of the right MTG and the left STG, left MTG, left PoCG and bilateral lenticular nucleus, putamen (PUT) (Figure 4A). Compared with women controls, women patients showed increased positive CBF connectivity between the seed ROI of the right ITG and the left ITG, bilateral superior frontal gyrus, orbital part (ORBsup). Moreover, women patients had increased positive CBF connectivity between the seed ROI of the right HIP and the temporal pole: middle temporal gyrus (TPOmid) and bilateral ITG (Figure 4B).

**TABLE 5:** Significant brain regions identified for ASL analyses of CBF connectivity.

ROI	Hemisphere	Brain regions	Peak MNI coordinates x, y, z (mm)	*t*-value	Voxels
**M patients vs. M controls**					
HIP.R	L	HIP	–30,–22,–16	–5.3211	116
MTG.R	L	STG	–60,4,–14	–5.775	543
	R	PUT	24,16,-8	–5.2988	36
	L	MTG	–64,-30,–6	–4.1474	69
	L	PUT	–22,16,6	–4.3148	27
	L	PoCG	–52,–8,36	–5.12	447
**F patients vs. F controls**					
HIP.R	L	ITG	–44,4,–34	4.3127	32
	R	TPOmid	46,6,–32	4.3267	30
	R	ITG	48,–22,–30	4.0515	31
ITG.R	L	ITG	–54,–28,–28	4.4699	54
	L	ORBsup	–14,50,–14	4.1241	13
	R	ORBsup	44,18,32	3.7749	15

M, Men; F, Women; R, Right; L, Left; HIP, Hippocampus; ITG, Inferior temporal gyrus; STG, Superior temporal gyrus; PUT, Lenticular nucleus, putamen; MTG, middle temporal gyrus; PoCG, postcentral gyrus; ORBsup, Superior frontal gyrus, orbital part; TPOmid, Temporal pole: middle temporal gyrus; ASL, Arterial-spin labeling; CBF, cerebral blood flow.

### Correlation analysis

**FIGURE 5:**
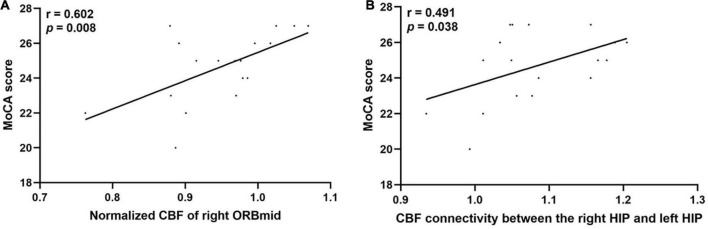
The statistically significant correlations between the normalized CBF and CBF connectivity changes and the MoCA score only in men patients. Spearman correlation analysis and Bonferroni correction for multiple comparisons were performed. *P* < 0.05 was considered statistically significant. **(A)** The MoCA score was positively correlated with the normalized CBF of the right ORBmid in men patients (*r* = 0.602, *P* < 0.05), N for Bonferroni corrections = 12; **(B)** The MoCA score was positively correlated with the CBF connectivity between the right HIP and the left HIP in men patients (*r* = 0.491, *P* < 0.05), N for Bonferroni corrections = 12. CBF, cerebral blood flow; MoCA, montreal cognitive assessment; ORBmid, middle frontal gyrus, orbital part; HIP, hippocampus.

For women patients, the normalized CBF of the right ITG was positively correlated with the naming score (*r* = 0.431, *p* = 0.045) and the normalized CBF of the right SFGdor was positively correlated with the abstraction score (*r* = 0.482, *p* = 0.023). For men patients, the normalized CBF of the right ORBinf were positively correlated with the orientation score (*r* = 0.468, *p* = 0.049). In addition, the normalized CBF of the right ORBmid were positively correlated with the MoCA score (*r* = 0.602, *p* = 0.008) (Figure 5A), the visuospatial/execution score (*r* = 0.495, *p* = 0.037), and the attention score (*r* = 0.538, *p* = 0.021). Furthermore, the CBF connectivity between the right HIP and the left HIP was positively correlated with the MoCA score (*r* = 0.491, *p* = 0.038) (Figure 5B) and the abstraction score (*r* = 0.484, *p* = 0.042) in men patients. The other regional CBF values and CBF connectivity were not correlated with the cognitive scores.

## Discussion

Consistent with former research which indicated an association between TBI and cerebrovascular dysfunction (Kisser et al., 2016; Wang et al., 2020), we found mTBI patients had lower normalized CBF in the ORBinf, ORBmid as well as the SFGdor, compared with controls. Frontal lobe was known to be vulnerable to mTBI (Li et al., 2019a; Lu et al., 2020). In mTBI, reduced neural activity was found in the inferior frontal gyrus, middle and superior frontal gyrus and these three parts tended to show decreased CBF (Wang et al., 2015; Churchill et al., 2017; Möller et al., 2017). Furthermore, the frontal gyrus plays a key role in cognitive function including working memory, language, attention and execution function (Li et al., 2017), thus, the hypo-frontality may contribute to the cognitive symptoms in mTBI (Dodd et al., 2020; Hamer et al., 2020). Our findings also suggested that cognitive impairment may be attributed to reduced perfusion in the frontal lobe. Our data additionally showed that men patients exhibited greater correlations between the normalized CBF of the frontal gyrus and cognitive function, indicating that normalized CBF changes are more likely to lead to cognitive impairment of men patients.

Previous studies have reported that mTBI patients exhibit diffused hippocampal and temporal neuronal damages (Lu et al., 2020). To our knowledge, the HIP which is located in the medial temporal lobe of brain is more vulnerable to brain injury (Vagnozzi et al., 2007; Monti et al., 2013). And neuroimaging studies showed signs of subtle vascular injury in the temporal lobe following TBI (Pöttker et al., 2017; Sullivan et al., 2021). Newberg et al. (2014) further found significantly decreased CBF in the right temporal lobe in mTBI patients by using SPECT. There were multiple interpretations for the observed effects of mTBI on CBF in men patients. These differences may be due to changes in the neurometabolic activity, along with potentially subtle reductions in cortical volume following brain injury, resulting in CBF demand reduction (Hamer et al., 2020). Additionally, the right MTG and the right HIP are connected with contralateral regions by the corpus callosum structurally and have inter-hemispheric connectivity, the right MTG has connection within temporal gyrus such as the STG (Li et al., 2019b). Moreover, the cortex of the temporal lobe is connected with the cortex of the parietal.

Interestingly, we revealed increased perfusion in the ITG and the HIP only in women patients following mTBI at the acute phase, which is inconsistent with one previous study (Bai et al., 2019). This inconsistency may be due to different study time after injury. Nevertheless, these two brain regions have been demonstrated volumetric atrophy in mTBI patients (Li et al., 2019b; Sampedro et al., 2020). One possible explanation for the increased normalized CBF values in the ITG and the HIP may be a short-term compensatory mechanism during the early stage following injury (Hamer et al., 2020). As aforementioned, the cortex of the temporal lobe is structurally connected with the cortex of the frontal and there are connections between the HIP and bilateral temporal lobes. These increased connections may be a compensatory response for lower normalized CBF in women patients.

The temporal gyrus is known to be a pathophysiological site of cognitive impairment which was caused at the early stages of disease (Mohamed et al., 2018). It is partially because of notable accumulations of amyloid in the temporal gyrus. The present study also showed that the CBF connectivity between the right HIP and the left HIP was positively correlated with the MoCA score and the abstraction score in men patients. The cognitive decline may be linked to hippocampal neurodegeneration in patients with mTBI which was confirmed by previous research (Melie-García et al., 2013; Zuo et al., 2019). However, women patients showed increased perfusion in these two brain regions. Increased perfusion in these two brain regions could produce or transport more metabolic substances which would improve cognitive function (Pöttker et al., 2017; Sullivan et al., 2021).

There were several limitations in our study. First, direct causal effects of sex differences cannot be inferred from this study because sex differences in normalized CBF and its connectivity are interacted by many factors. Second, the sample size was small, despite the clear findings, their replication in a larger sample is mandatory. Third, while this study aimed to examine cognitive decline in mTBI at the acute stage, imaging data was only acquired once within 14 days following brain injury. No longitudinal data were available at additional time points for studying the progression of recovery. Finally, no other studies looked at sex specific changes relating to a more severe injury. Thus, further prospective studies are warranted to investigate if there are similar sex differences in CBF with a repetitive mild TBI or more severe TBI.

## Conclusion

The current study demonstrated sex differences in normalized CBF and CBF connectivity in some cortical and subcortical regions in acute mTBI patients, which would help to understand the neurobiological mechanism of sex-specific effects of mTBI on brain physiology. This study also observed an association between CBF and CBF connectivity alterations and cognitive function, which would further to understand the pathophysiology of sex-specific cognitive impairment after acute mTBI.

## Data availability statement

The original contributions presented in this study are included in the article/supplementary material, further inquiries can be directed to the corresponding authors.

## Ethics statement

The studies involving human participants were reviewed and approved by the Institutional Review Board of Nanjing Medical University. The patients/participants provided their written informed consent to participate in this study.

## Author contributions

MD and YL drafted the manuscript for the work. FL and LL helped to acquire the clinical and fMRI data. Y-CC did the financial support, review, and final approval of the manuscript to be published. All authors have read and approved the final manuscript.

## Conflict of interest

The authors declare that the research was conducted in the absence of any commercial or financial relationships that could be construed as a potential conflict of interest. The handling editor VM and reviewer ZX declared a past co-authorship with the authors Y-CC, LL, and FL.

## Publisher’s note

All claims expressed in this article are solely those of the authors and do not necessarily represent those of their affiliated organizations, or those of the publisher, the editors and the reviewers. Any product that may be evaluated in this article, or claim that may be made by its manufacturer, is not guaranteed or endorsed by the publisher.

## Footnotes

1
http://www.fil.ion.ucl.ac.uk/spm

2
https://cfn.upenn.edu/

3
http://rfmri.org/
